# Is More Better? Benefits of Hyaluronic Acid Soft Tissue Filler on the Psychological- and Social-Related Quality of Life Dimensions

**DOI:** 10.1093/asjof/ojac086

**Published:** 2022-11-17

**Authors:** Alain Michon, Haidar Hassan

**Affiliations:** Medical director of a private aesthetic practice, Ottawa, Canada; Clinical senior lecturer, Blizard Institute, Queen Mary University of London, London, UK

## Abstract

**Background:**

Hyaluronic acid (HA) soft tissue fillers are popular for volumizing, sculpting, or rejuvenating the face. Their effect beyond these cosmetic benefits remains poorly defined, especially the changes in the psychological and social dimensions of health following cosmetic HA filler injections.

**Objectives:**

To determine whether injecting more facial aesthetic units with HA soft tissue filler will improve the health-related quality of life dimension of patients.

**Methods:**

A prospective study was conducted to assess the psychological and social benefits of treating multiple facial aesthetic units with HA soft tissue filler using 3 validated FACE-Q scales at baseline and 4 weeks posttreatment between January and August 2022.

**Results:**

Data for 28 participants (*n* = 26 females [93%]; mean age: 49.7 ± 10.1 years) are available and reveal significant improvements on the psychological (+24.5; *P* < .001) and social functioning (+18.5; *P* < .001) FACE-Q scales and a reduction in the appearance-related distress score (−17.8; *P* < .001) posttreatment compared to baseline. A mean volume of 4.7 mL (range 1.0-15.2 mL) was injected. However, patients who had >2 facial aesthetic units injected did not statistically score better on the FACE-Q scales.

**Conclusions:**

Facial treatment with HA fillers was associated with a marked improvement in the health-related quality of life dimension. While understanding patients’ aims and motivation, an individualized treatment approach is strongly encouraged instead of a “whole-face” approach.

**Level of Evidence: 3:**

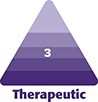

A plethora of processes are involved in facial aging, and over time these changes will result in bone remodeling, deflation and augmentation of discrete fat compartments, loss of ligamentous support, and alteration of facial muscle activity. Superficially, the skin will also exhibit some modifications, including skin atrophy and loss of elasticity and hydration, and in combination with subdermal changes, they will induce visible changes altering the facial appearance.^1–4^ The aging phenotype is often associated with negative emotions for the person growing old, including a poor function of the psychological and social dimension of health, as well as being seen as less attractive by the perceivers.^5,6^ Hence, the field of aesthetic medicine is gaining popularity in the seeking of rejuvenation procedures.^7^

Without any doubt, facial appearance is closely related to the overall perceived physical attractiveness. This perception, embedded in our genetics, starts early in life, as demonstrated in 6-month-old infants who prefer attractive faces.^8^ Attractiveness and beauty have strong psychological and social implications. For example, beautiful children are treated more positively, and attractive adolescents are shown to be more popular than less attractive classmates, independent of their academic performance.^9,10^ When asking for help, the more attractive one is, the more likely one will receive support when asked for it.^11^ In the work environment, it was demonstrated that the more attractive a person is, the more likely he or she will be employed and have higher wages and premiums on average.^12^ This is described as the halo effect, demonstrated in all ages and races, where positive traits are given to people ranking higher on the attractive scale.^10^ First impressions are, therefore, closely related to perceived beauty. They are vital as they affect the reactions and decisions toward a person and will exhibit some influence on how this person will counter react.

Newer soft tissue fillers made of hyaluronic acid (HA) with different characteristics and rheology made their debut in the 21st century.^13^ These novel products confer practitioners increased versatility and indications for use in facial aesthetics. Not only can HA fillers be used to decrease folds or correct lines, but they are now utilized alone or in combination for treating any facial aesthetic units and improving the overall facial appearance for optimal outcomes.^6^ These indications and end goals include but are not limited to facial contouring, shaping, volumizing, hydration, myomodulation, reducing folds, and wrinkles, and improving facial symmetry, ratio, and harmony.^14,15^ This is now possible with HA soft tissue fillers, and they may be used to their full potential for complete facial rejuvenation and improving attractiveness.

Until recently, randomized controlled trials have mainly looked at the efficacy of HA fillers in correcting folds or improving facial appearance.^16^ More specifically, the psychological and social benefits seen in persons who received HA fillers were rarely assessed, although crucial as it may lead to better functioning of the individual.^17^ There is a need to understand better the roles of HA soft tissue fillers on the psychological and social dimensions of health. It is unknown if injecting more facial aesthetic units with HA soft tissue filler on patients will generate a more significant psychosocial impact. This study aims to determine whether injecting more facial aesthetic units with HA soft tissue filler will improve the health-related quality of life dimension of patients.

## METHODS

### Study Design

This is a prospective single-center clinical study with a start date of January 2022 and an end date of August 2022 on patients who presented for HA soft tissue filler treatment for aesthetic purposes. The study received approval from the research ethics committee of the institution du Savoir Montfort, study # 21-22-11-027; and is conducted in accordance with the good clinical practice and regional laws and regulations, and adheres to the Declaration of Helsinki principles.

### Population Study

Twenty-eight participants, 26 females (93%) and 2 males (7%), with a mean age of 49.7 ± 10.1 years, who presented in a private aesthetic practice in Ottawa, Canada for HA filler injections were recruited. Written consent was obtained for all agreeing participants. Then, validated FACE-Q questionnaires (psychosocial function, appearance distress, and social function) on the health-related quality of life dimension were given to participants and collected before and 4 weeks after treatment. The inclusion criteria for the participants were as follows:

- adults between 30 and 65 years of age;
- BMI below 30 kg/m^2^;
- absence of soft tissue filler, neuromodulator and biostimulator agents, threads, and suspension sutures treatments of the face in the past year;
- absence of energy-based devices and chemical peels for facial rejuvenation in the past year;
- absence of previous neck or facial surgery;
- absence of allergies, contraindications, comorbidities, or chronic diseases that exclude the injections of soft tissue fillers in the face;
- able to provide written informed consent to participate in the study, including the use of personal data for research purposes; and
- does not meet the criteria for body dysmorphic disorder—assessed with the use of a cryptic screening protocol and during the consultation.

Patients were excluded from the study for any of the following reasons:

- does not meet inclusion criteria;
- unable to read in English or French;
- unable to consent; and
- unwillingness to follow up.

### FACE-Q Questionnaires

The FACE-Q aesthetics module comprises of independently validated psychometric and easy-to-use patient-reported outcome measures that can be used to measure outcomes for any type of cosmetic treatment.^18–21^ Three domains are available. The study focuses on the domain of health-related quality of life, measuring changes with the following scales:

- Psychological Function: This 10-item scale measures psychological function. Items ask respondents to answer with their facial appearance in mind. The questions ask about feeling happy, attractive, confident, and good about oneself.
- Appearance Distress: This 8-item scale measures appearance-related distress in people seeking cosmetic treatments for the body or the face. The questions ask someone to agree/disagree with statements about feelings (eg, unhappy, stressed, and down) and behaviors, such as avoiding being around people.
- Social Function: This 8-item scale measures social function. Items ask respondents to answer with their facial appearance in mind. Questions ask about feeling confident when meeting new people, in new social situations, and when participating in group situations.

Items for each questionnaire were answered using a 4-point scale: (1) definitely disagree; (2) somewhat disagree; (3) somewhat agree; and (4) definitely agree.

### Facial Aesthetic/Units

**Figure 1.**
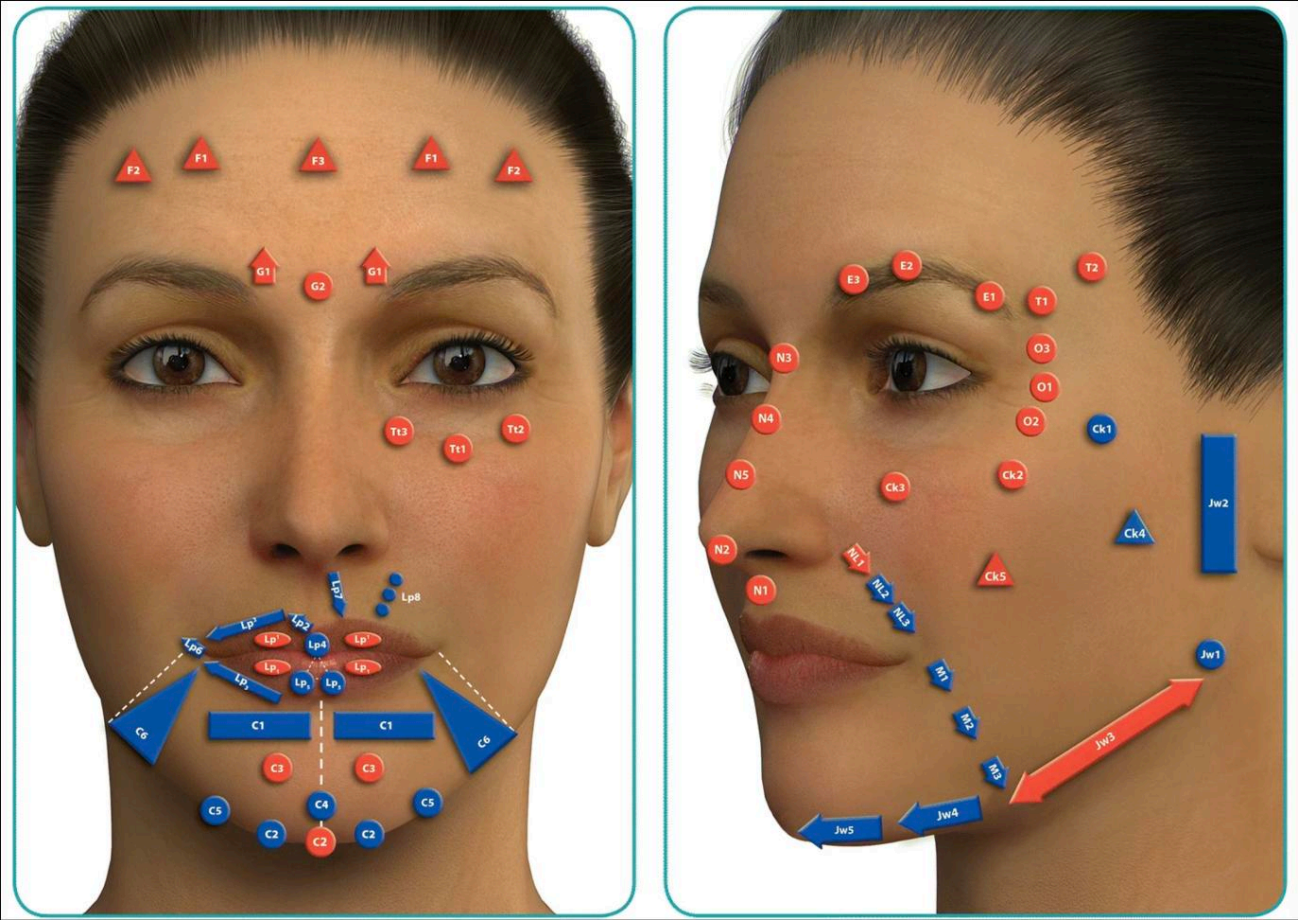
Picture depicting facial aesthetic units and their associated subunits, as per the MD codes system for cosmetic injectors.^23^ The 12 facial units are F: forehead; T: temple; E: eyebrow; G: glabella; O: lateral orbital; Tt: tear trough; Ck: cheek; NL: nasolabial fold; Lp: lip; M: marionette lines; C: chin; Jw: jawline. Images developed in connection with Dr Mauricio de Maio's MD Codes system and reprinted with permission from Allergan Aesthetics, an AbbVie company, Dublin, Ireland.

The face can be divided into specific areas, designated as “aesthetic units” (Figure 1), within which the skin has similar characteristics.^22,23^ These characteristics include skin color, thickness, amount of subcutaneous fat, texture, and presence of hair. These “units” are separated from each other by relatively well-defined ridges and creases, designated as “aesthetic borders.” The borders include easily discernable landmarks such as the hairline, eyebrows, nasolabial fold, philtrum, vermillion border, and labiomental fold.^24^ Due to the worldwide knowledge of the MD codes system for cosmetic injectors, we used the definition of facial units by de Maio^23^ in the study.

### Treatment Techniques

After a thorough consultation with the patients, treatment plans were developed based on their individual motivations, aims, and needs. Patients requiring dynamic wrinkle injections, or other aesthetic treatments, had these procedures delayed until the HA filler follow up session and questionnaires were obtained.

Patients were treated with injectable HA filler from the Juvederm brand (Allergan Aesthetics, an AbbVie company, Irvine, CA), including the Vycross range (Volbella, Volift, Voluma and Volux) and Hylacross range (Ultra or Ultra Plus), and are approved for cosmetic use in Canada. The skin was make-up free and cleaned thoroughly with a solution of Chlorhexidine 2% w/v aqueous and Isopropyl alcohol 4% v/v (Omega Laboratories Limited, Canada) and left to dry prior to injections. HA filler was either injected via a needle or blunt microcannula (TSK Laboratory, Japan), depending on the clinical indications and results looking to be achieved. Needle aspiration was performed for each injection using a 27G or 30G sharp needle.

### Study Assessments

Baseline demographic of patients was collected, including pre- and postphotographs and a detailed record of the injectable treatment session. Participants filled up the 3 FACE-Q scales the day of, before the procedure, and at the 4-week follow-up visit, which was booked at check-out, as treatment was not free of charge. No touch-up treatment was permitted until then. An appointment text messaging reminder was sent 1 week prior to the 4-week follow-up session.

For each scale, the sum scores were calculated as totals out of 32 or 40. The scores were then converted into “Rasch” scores out of 100, as per instruction for each FACE-Q questionnaire.

### Statistical Analysis

Changes in the Rasch score for each Face-Q questionnaire were analyzed using a paired *t*-test and a statistical significance of *P* < .05. The effect of treating 1 or 2 vs 3 or more facial aesthetic units with HA fillers was also analyzed.

Descriptive statistics are provided for all variables. Mean and SD were used for continuous variables, and frequency and percentage for categorical variables. Paired sample *t*-test was performed to compare the difference between pretreatment and posttreatment on FACE-Q quality of life questionnaire Rasch scores. Pearson correlations were performed for associations between the number of areas injected and FACE-Q quality of life questionnaire Rasch scores (pre- and posttreatment).

Finally, independent-sample *t*-tests were conducted to compare FACE-Q quality of life questionnaire Rasch scores posttreatment between 1 and 2 areas vs 3+ areas injected. Data were analyzed using IBM SPSS Statistics for Windows, Version 22.0 (Armonk, NY).^25^ A *P*-value of .05 was used to indicate statistical significance.

## RESULTS

### Participants

In total, 30 participants were enrolled from January to August 2022, and 93.3% completed the study. Two patients were considered lost to follow up, as they did not show up to their follow-up visit, despite text messaging reminders and not responding to follow-up calls.

Completed data are available for the 28 patients who have completed the study, of whom 26 (93%) were females, and 2 (7%) were males, with a mean age of 49.7 ± 10.1 years (range 33-69; Table 1). The mean amount of HA filler injected was 4.7 ± 3.6 mL (range 1.0-15.2), and 32% of patients had 1 or 2 facial aesthetic units injected, whereas 68% had injections in 3 or more facial aesthetic units.

**Table 1.** Patient Characteristics (*N* = 28)

Characteristics	1-2 aesthetic unit (*n* = 9)	≥3 aesthetic units (*n* = 19)	Overall (*n* = 28)
Gender, *n* (%)			
Women	9 (100.0%)	17 (89.5%)	26 (93%)
Men	0 (0.0%)	2 (10.5%)	2 (7%)
Age, mean ± SD (range)	53.7 ± 10.1	47.8 ± 9.87	49.7 ± 10.1 (33-69)
Amount injected, mean ± SD	1.74 ± 0.97	6.14 ± 3.50	4.7 ± 3.6 (1.0-15.2)

Descriptive statistics are provided throughout, mean and SD were used for continuous variables, and frequency and percentage for categorical variables. SD, standard deviation.

### Patient-Reported Outcomes

**Figure 2.**
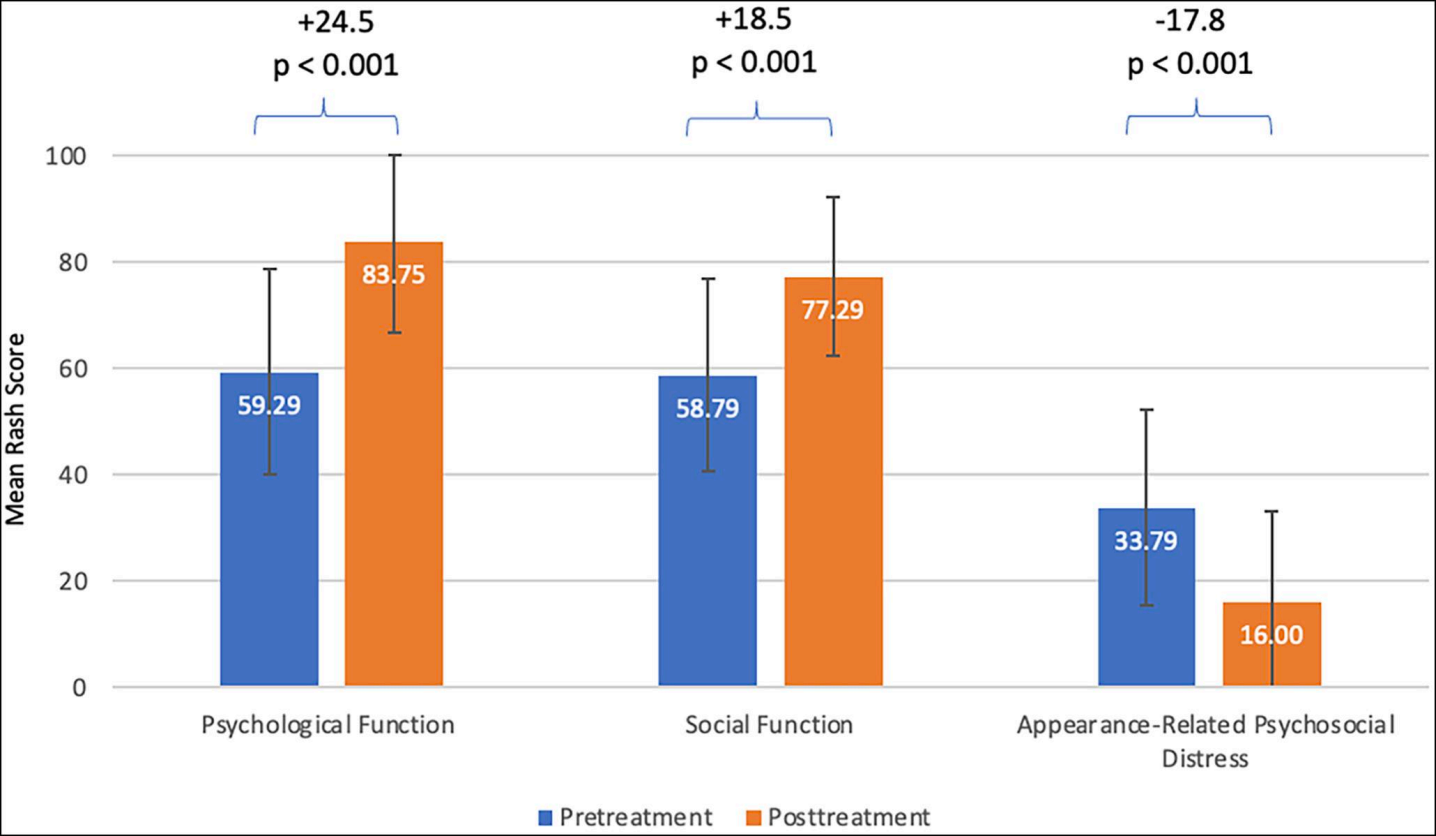
FACE-Q quality of life questionnaire scores (*N* = 28).

There is a statistically significant improvement after treatment in all three FACE-Q scales from baseline pretreatment results (Figure 2). Paired sample *t*-test was performed to compare the difference between pretreatment and posttreatment on FACE-Q quality of life questionnaire scores. Psychological function score increased from 59.29 ± 19.25 to 83.75 ± 17.00 (*P* < 0.001), with a mean change of +24.5 posttreatment. Social function increased from 58.79 ± 18.24 to 77.29 ± 15.00 (*P* < 0.001), with a mean change of +18.5 posttreatment. Appearance-related psychosocial distress score decreased from 33.79 ± 16.00 to 18.44 ± 17.09 (*P* < 0.001), with a mean change of −17.8 posttreatment.

### Facial Aesthetic Units and FACE-Q Scores

An independent-samples *t*-test was conducted to compare FACE-Q quality of life questionnaire scores posttreatment between 1 and 2 facial aesthetic units vs 3 or more facial aesthetic units injected (Table 2). There were no significant differences in the FACE-Q quality of life questionnaire scores between the two groups. These results suggest that the number of facial aesthetic units injected does not have an effect on the FACE-Q quality of life questionnaire scores in patients.

**Table 2.** Independent-Sample *t*-Test Comparison Between the Number of Aesthetic Units Injected on FACE-Q Quality of Life Questionnaire Scores (*N* = 28)

Scale	No. of aesthetic units injected	*N*	Mean Rasch score	SD	*P*-value
Social function posttreatment	1-2 unit	9	80.00	17.916	.520
	3+ units	19	76.00	13.764	
Appearance-related psychosocial distress posttreatment	1-2 unit	9	15.78	20.092	.963
	3+ units	19	16.11	16.079	
Psychological function posttreatment	1-2 unit	9	86.89	19.284	.512
	3+ unis	19	82.26	16.155	

SD, standard deviation.

Furthermore, a Pearson correlation was performed to see if there were associations between the number of facial aesthetic units injected and FACE-Q quality of life questionnaire scores (pre- and posttreatment). Results of the Pearson correlation indicated that there were no significant associations between the number of areas injected for all FACE-Q quality of life questionnaire scores (Table 3). An increase in the number of injected areas is therefore not associated with an increase nor a decrease in the FACE-Q quality of life questionnaire scores.

**Table 3.** Pearson Correlation Between FACE-Q Quality of Life Questionnaire Scores and the Number of Facial Aesthetic Units Injected (*N* = 28)

Scale	Pearson correlation
Social function pretreatment	−0.065 (*P* = .741)
Social function posttreatment	−0.031 (*P* = .876)
Appearance-related psychosocial distress pretreatment	−0.013 (*P* = .948)
Appearance-related psychosocial distress posttreatment	−0.0.68 (*P* = .733)
Psychological function pretreatment	0.009 (*P* = .966)
Psychological function posttreatment	0.107 (*P* = .589)

### Safety

Although a detailed safety data are unavailable for the current study, none of the patients reported ongoing adverse events at the 4-week follow-up visit. Common transient treatment-related events reported were firmness, tenderness to touch, and swelling at the site of injections.

## DISCUSSION

Results of this prospective clinical study confirm that injectable cosmetic treatments with HA soft tissue fillers in a typical aesthetic practice were associated with remarkable improvements in patients’ psychological and social functions, as well as a considerable reduction in their appearance-related distress using the validated FACE-Q health-related quality of life dimension scales. Many previous studies have failed to demonstrate the effect of HA fillers on the psychological and social health of patients, the vast majority focusing solely on patients’ satisfaction with the treatment results and first impressions.^26,27^ Only 1 study looked at the effect of cosmetic injectables (onabotulinumtoxin A and HA fillers) on the health-related quality of life dimension.^17^ However, the authors did not look specifically at the effect of HA fillers on these scales. To the authors’ knowledge, there are no previous studies evaluating the impact of injecting multiple facial aesthetic units with HA fillers on the psychological and social well-being of patients.

The benefits of HA filler treatments are greater on the psychological function, with a mean change of +24.5 from baseline, compared to social function (mean change of +18.5) and appearance-related psychosocial distress (mean change of −17.8). These results are different from the observation by Mckeown and could be due to the fact that the patients enrolled in the present study scored less on the appearance-related distress scale at baseline.^17^ However, this is not surprising given that many of the patients expressed unhappiness with one or more of the facial aesthetic units treated. The psychological function scale looks at happiness, attractiveness, confidence, etc, and is closely related to facial appearance, aligned with current evidence.^28–32^ Also, changes in the psychological and social function scales are more significant than previously reported with cosmetic injectables, likely because this study focused on HA fillers and resulted in a more remarkable change in facial appearance compared to onabotulinumtoxin A injections.^17^ Furthermore, although the comparison is difficult between studies, the changes on each of the three FACE-Q scales were aligned with previous results after plastic surgery.^33,34^

Surprisingly, results of the independent-samples *t*-test and the Pearson correlation confirm that there were no significant differences in the scores for all three FACE-Q scales on the health-related quality of life dimension between patients who had 1 or 2 facial aesthetic units injected vs 3 or more aesthetic units, and those who had more aesthetic units injected. Recently, many authors have given attention to the “whole-face approach” with HA fillers and treating emotional attributes to improve aesthetic outcomes, including patient satisfaction.^23,35,36^ However, a retrospective charts review revealed that all patients who had 1 or 2 aesthetic units treated were focused on improving a specific facial area, that is, lips or undereye hollowness, and were not at all concerned nor wanted to improve other facial areas or emotional attributes (being sad, tired, saggy, or angry looking). Conversely, treating emotional attributes raised during the consultation process and in selected patients led to positive outcomes on all three Face-Q scales used in the present study. This confirms the importance of truly understanding the patients’ motivations and aims, independently of the subjective perceived needs and that the “whole-face approach” will not necessarily translate into a more significant impact on the health-related quality of life dimension of patients. Having a better understanding of the patient's motivations was also recommended by Liew et al.^37^ Based on the data analysis of 54,000 participants from 17 different countries, they categorized patients into four archetypes and favored an individualized treatment plan and guidance based on patients’ motives. Having the patient's best interest should be a priority for all aesthetic practitioners.

It is important to note that the effect of HA filler on the psychosocial function and appearance-related distress is likely underestimated due to the COVID-19 pandemic. The study started in January 2022 and was affected by lockdowns and ongoing restrictions, interfering negatively with the day-to-day activities and social life of participants, including the enrollment and completion of the study. The pandemic could have also played a role in the two patients who were lost in follow up.

In addition, other limitations of the study are: (1) the FACE-Q questionnaires were completed before and 4 weeks after treatment, and the long-term effect of HA fillers on the health-related quality of life is unknown, including if repeated or treating additional aesthetic units would result in a higher score; (2) the study, although statistically significant, has a small number of participants and could also induce placebo effect being a single-arm study; (3) this is a single-center study, and a larger randomized, multi-center controlled trials would provide valuable higher evidence data.

## CONCLUSIONS

Currently, most aesthetic medicine injectable studies have focused on the efficacy of HA dermal fillers volumizing or correcting an area of the face, neglecting necessary patient-reported psychological and social outcome measures. However, results of this prospective clinical study indicate that HA filler used in facial aesthetics significantly improved patients’ psychological function, appearance-related psychosocial distress, and social function, as measured on the FACE-Q scales at baseline and one month after the injectable procedure. Interestingly, all three scales’ scores were not greater in patients with more facial aesthetic units treated with HA fillers. Hence, the “whole-face” approach may not be as important as understanding a patient's specific motivations and aims when looking primarily at HA filler and its effect on the psychological and social dimensions of health. Also, the results may be underestimated due to the COVID-19 pandemic and associated societal restrictions affecting the day-to-day life of the study participant. There is a need for more extensive studies with a control group and longer follow ups to confirm the tremendous benefits of HA fillers on the health-related quality of life dimension, which may be beyond treating wrinkles and folds.
